# Leptin Modulates the mRNA Expression of Follicle Development Markers in Post-hatch Chicks in an Age-Dependent Manner

**DOI:** 10.3389/fphys.2021.657527

**Published:** 2021-07-07

**Authors:** Amir Hossan Shaikat, Masami Ochiai, Akari Sasaki, Misa Takeda, Akari Arima, Takeshi Ohkubo

**Affiliations:** ^1^College of Agriculture, Ibaraki University, Ami, Japan; ^2^United Graduate School of Agricultural Science, Tokyo University of Agriculture and Technology, Fuchu, Japan

**Keywords:** chick, development, follicle, leptin, ovary

## Abstract

Leptin is involved in regulating reproductive function in chickens, and the development of the leptin system is initiated during the early embryonic stage; however, whether leptin has a specific role in regulating the ovarian development in early post-hatch days is still not fully understood. This study investigated the expression of ovarian functional markers in growing juvenile chickens, along with the effects of leptin on gene expression in the hypothalamus–pituitary–gonadal (HPG) axis on specific ovarian-remodeling days. Leptin receptor (LEPR), follicle-stimulating hormone receptor (FSHR), and the mRNA expression of aromatase (CYP19A1) tended to increase with age in the ovaries of growing chicks. In the ovaries of 7-day-old chicks, intraperitoneally injected leptin significantly increased the mRNA expressions of LEPR, FSHR, and CYP19A1, and this resulted in the increased serum estradiol levels. However, leptin had no effect on hypothalamic LEPR, gonadotropin-releasing hormone 1 (GnRH1), or gonadotropin-inhibitory hormone (GnIH) mRNAs; however, in the pituitary gland, leptin significantly increased the mRNA expression of luteinizing hormone beta subunit (LHB) but had no effect on the mRNA expression of follicle-stimulating hormone beta subunit (FSHB). In 28-day-old chicks, hypothalamic and pituitary mRNAs were unaffected by leptin administration, except hypothalamic LEPR mRNA that was upregulated by a high dose of leptin. In the ovary, leptin dose-dependently decreased the mRNA expression of LEPR; low doses of leptin significantly increased the mRNA expression of FSHR, whereas high doses significantly decreased this expression; leptin did not affect the mRNA expression of CYP19A1; and high leptin doses significantly reduced the serum estradiol levels. Collectively, the results of this study show that leptin modulates ovarian development and folliculogenesis marker genes by primarily acting on ovaries on the specific ovarian-remodeling days in post-hatch chicks, which may alter folliculogenesis and ovarian development toward puberty in chicken.

## Introduction

The hypothalamus–pituitary–gonadal (HPG) axis is involved in the ovarian development, and gonadotropins, sex steroid hormones, and other factors modulate the ovarian function directly or indirectly. Among these factors, leptin, an adipokine identified as a molecule that plays a role in obesity, affects the ovarian function through the hypothalamo-hypophyseal system and/or directly through the gonads in vertebrates. Leptin indirectly regulates gonadotropin-releasing hormone (GnRH) neuron activity through the forebrain in mice (Quennell et al., 2009), even though GnRH neurons do not express leptin receptors (LEPRs) (Finn et al., 1998; Hâkansson et al., 1998). In addition, leptin has a specific role in the onset of puberty through the melanocortinergic system (Manfredi-Lozano et al., 2016; Egan et al., 2017). In chickens, there is evidence that the continuous leptin administration raises the levels of gonadotropins and sex steroid hormones, resulting in the advanced onset of puberty (Paczoska-Eliasiewicz et al., 2006), and leptin prevents starvation-induced apoptosis in the follicular wall of F1–F3 follicles, which ameliorates the fasting-induced cessation of egg laying (Paczoska-Eliasiewicz et al., 2003). Furthermore, leptin improves the follicular hierarchy dysfunction observed in *ad libitum*-fed broiler breeders (Cassy et al., 2004). Leptin also affects ovarian steroidogenesis since it promotes the release of estradiol in cultured chicken ovarian cells (Sirotkin and Grossmann, 2007) and goose granulosa cells (Hu et al., 2014). Moreover, an increase in estrogen release by leptin improves the egg-laying record in restricted-fed laying hens (Sirotkin and Grossmann, 2015). These results suggest that leptin contributes, at least in part, to ovarian development and folliculogenesis in birds. The development of the leptin system is started during the embryonic phase in chickens because leptin and the mRNA expression of LEPR have been detected in the ovaries of chick embryos after 4.5 days of incubation (Seroussi et al., 2016), and the LEPR mRNA is expressed in the ovaries throughout the second half of the embryonic phase (Shaikat et al., 2018). Leptin advances the day of first egg laying when it injected *in ovo* in the Japanese quail (Lamošová et al., 2003). Collectively, leptin may modulate the follicle development and ovarian function from their early embryonic phase to adulthood in birds. Ovarian differentiation in most birds starts when the cortex develops in the left gonad in the early embryonic phase (Smith and Sinclair, 2004) and germ cells undergo mitosis or meiosis under control of gonadotropin in the embryonic ovary (He et al., 2013). After hatching, the formation of primordial follicles (i.e., initial recruitment) occurs within the ovarian cortex at around 1 week of age (González-Morán, 2007, 2011; Johnson, 2015), and the transition from primordial follicles to primary follicles begins at 4 weeks of age (Johnson and Woods, 2007; González-Morán, 2011; Johnson, 2014). These processes play a vital role in the development of ovarian function toward sexual maturity in chickens, and that is suggested to be regulated by several hormones. Although a lot of evidence indicates that leptin shows a positive effect on puberty in vertebrates, most leptin studies on ovarian development and folliculogenesis in birds have been focused on the prepubertal stage and after puberty in associating with the laying cycle, and available evidence of early post-hatch regulation of the ovarian development by leptin is limited.

In this regard, this study aimed to investigate the role of leptin in regulating the follicle development in juvenile chickens. To this end, the post-hatch changes in the mRNA expression of follicle-stimulating hormone receptor (FSHR), CYP19A1, and LEPR were analyzed in growing chicks. In addition, the effects of leptin in the ovaries, on specific ovarian-remodeling days, were investigated by analyzing the mRNA expression of functional markers in the HPG axis and LEPRs.

## Materials and Methods

### Animals

Newly hatched White Leghorn female chicks were obtained from Japan Layer K. K. (Gifu, Gifu, Japan). The birds were maintained with 24-h light at 30°C for the first 2 weeks and 23-h light at 29°C thereafter. All birds had free access to commercial chick feed (crude protein 21% and metabolizable energy 2,900 Kcal/kg; FEED ONE, Kanagawa, Japan) and water. The Ibaraki University Animal Care and Use Committee approved all animal care and procedures (Certification No. 17110).

For an ontogenic experiment, four chicks each from ages 1, 7, 14, 21, and 28 days old were sacrificed by cervical dislocation followed by dissection at the atlanto-occipital joint, and the left ovary was removed. The excised ovary was immediately snap-frozen in liquid nitrogen and stored at −80°C until use.

For a leptin administration study, recombinant mouse leptin (rmleptin; Sigma–Aldrich Japan, Tokyo, Japan) was used in this study, because genuine recombinant chicken leptin derived from recently cloned cDNA (Farkašová et al., 2016; Seroussi et al., 2016) may not be available. On the other hand, rmleptin remains biologically active in chicken and other birds and is used in ovarian function and other studies (Bungo et al., 1999; Máčajová et al., 2002, 2003; Lamošová et al., 2003; Song et al., 2009; Adachi et al., 2012; Hu et al., 2012; Niu et al., 2019). The rmleptin was dissolved in phosphate-buffered saline [PBS (–)] and injected intraperitoneally (i.p.) into six chicks each of 7 and 28 days old at doses of 0, 25, or 250 μg/kg body weight (BW). Of note, 24 h after injection, blood was collected by direct heart puncture, birds were killed by cervical dislocation followed by decapitation, and their hypothalamus, pituitary gland, and ovary were all collected. All tissues were immediately snap-frozen in liquid nitrogen and stored at −80°C until use. The serum was separated from coagulated blood, by centrifugation at 3,000 rpm for 30 min and stored at −80°C for further analysis. The same experiment has been conducted two times to confirm the reproducibility in the administration study.

### Reverse Transcription-Quantitative Real-Time PCR (RT-qPCR) Analysis

Total RNA was extracted from tissues using ISOGEN II (Nippon Gene, Toyama, Japan) according to the protocol of the manufacturer, and the RNA concentration was measured by a spectrophotometer (BioSpec-nano, Shimadzu, Kyoto, Japan). Subsequently, 400 ng of total RNA was reverse-transcribed using ReverTra Ace qPCR RT Master Mix with a gDNA remover (TOYOBO, Osaka, Japan). The reverse transcription was performed at 37°C for 15 min, then at 50°C for 5 min, followed by heating at 98°C for 5 min to terminate the reaction. The reverse-transcribed samples were subjected to the qPCR analysis. In Experiment 1, the mRNA expressions of LEPR, FSHR, and CYP19A1 was analyzed. In Experiment 2, the following were investigated: hypothalamic gonadotropin-releasing hormone 1 (GnRH1), gonadotropin-inhibitory hormone (GnIH), and LEPR mRNAs; pituitary follicle-stimulating hormone beta subunit (FSHB) and luteinizing hormone beta subunit (LHB) mRNAs; and ovarian LEPR, FSHR, CYP19A1, and suppressor of cytokine signaling 3 (SOCS3) mRNAs. The chicken ribosomal protein S17 (S17) was chosen as a reference gene to normalize the expression of target genes. The sequence of primers used in qPCR is listed in Table 1. The qPCR was performed using GoTaq qPCR Master mix (Promega, Madison, WI, USA) according to the protocol of the manufacturer on a TaKaRa RealTime Thermal Cycler Dice II (TaKaRa Bio, Shiga, Japan). The qPCR parameters were as follows: initial denaturation at 95°C for 10 s, followed by 40 cycles of 95°C for 5 s and of 60°C for 30 s. The relative quantification of the target and reference genes was evaluated according to standard curves. The relative gene expression was calculated using the calibration curve method by duplicates in each sample, and the values were expressed as means ± SEM.

**Table 1:** List of primers used for real-time PCR.

**Gene**	**Accession no**.	**Sequence (5**^****′****^**-3**^****′****^**)**		**Amplicon (bp)**
		**Forward**	**Reverse**	
GnRH1	NM001080877.1	ACACTGGTCTTATGGCCTGCA	ATTCAGCCTTCTGCCCTTCTC	116
GnIH	NM204363.1	GCATGGTATGTGCCTAGATGAACTAAT	TCCTCTGCTTTTCCTCCAAGATA	110
FSHB	NM204257.1	CCACGTGGTGCTCAGGATACT	AGGTACATATTTGCTGAACAGATGAGA	84
LHB	HQ872606.1	AACGTAACGGTGGCGGTG	AGGCCGTGGTGGTCACAG	64
LEPR	NM204323	TCTGCTCAGAGGTGTGGGAT	CTGAAACTGCGGCACGTATG	103
FSHR	NM205079.1	ACCTGCCTGGATGAGCTAAAT	ATCCAAAACAACAGGCCCGA	96
CYP19A1	NM001001761.2	CCAGTTGCCACAGTGCCTAT	CCTGGCCCTGGTATTGATGA	89
S17	NM204217.1	GACCCGGACACCAAGGAAAT	GCGGCGTTTTGAAGTTCATC	100

### Enzyme-Linked Immunosorbent Assay (ELISA) for Estradiol

Serum estradiol was measured using a commercial estradiol ELISA kit (Cayman Estradiol ELISA kit code 582251, Cayman, Ann Arbor, Michigan, USA). All the serum samples were run in duplicate. The assay sensitivity of the ELISA kit was 6.6–4,000 pg/ml.

### Statistical Analysis

All results obtained in this study, i.e., qPCR and plasma estrogen levels, were applied to the Smirnov–Grubbs test to exclude outliers and followed by Bartlett's test to determine homoscedasticity. Based on the result from Bartlett's test, the Tukey HSD (honestly significant difference) test and the Steel–Dwass test were used for the parametric and non-parametric analyses, respectively. All the statistical analyses were performed by R statistical package (www.r-project.org).

## Results

### Ontogenetic Changes of LEPR and Ovarian Functional Parameters in the Ovary of Juvenile Chicks

**Figure 1:**
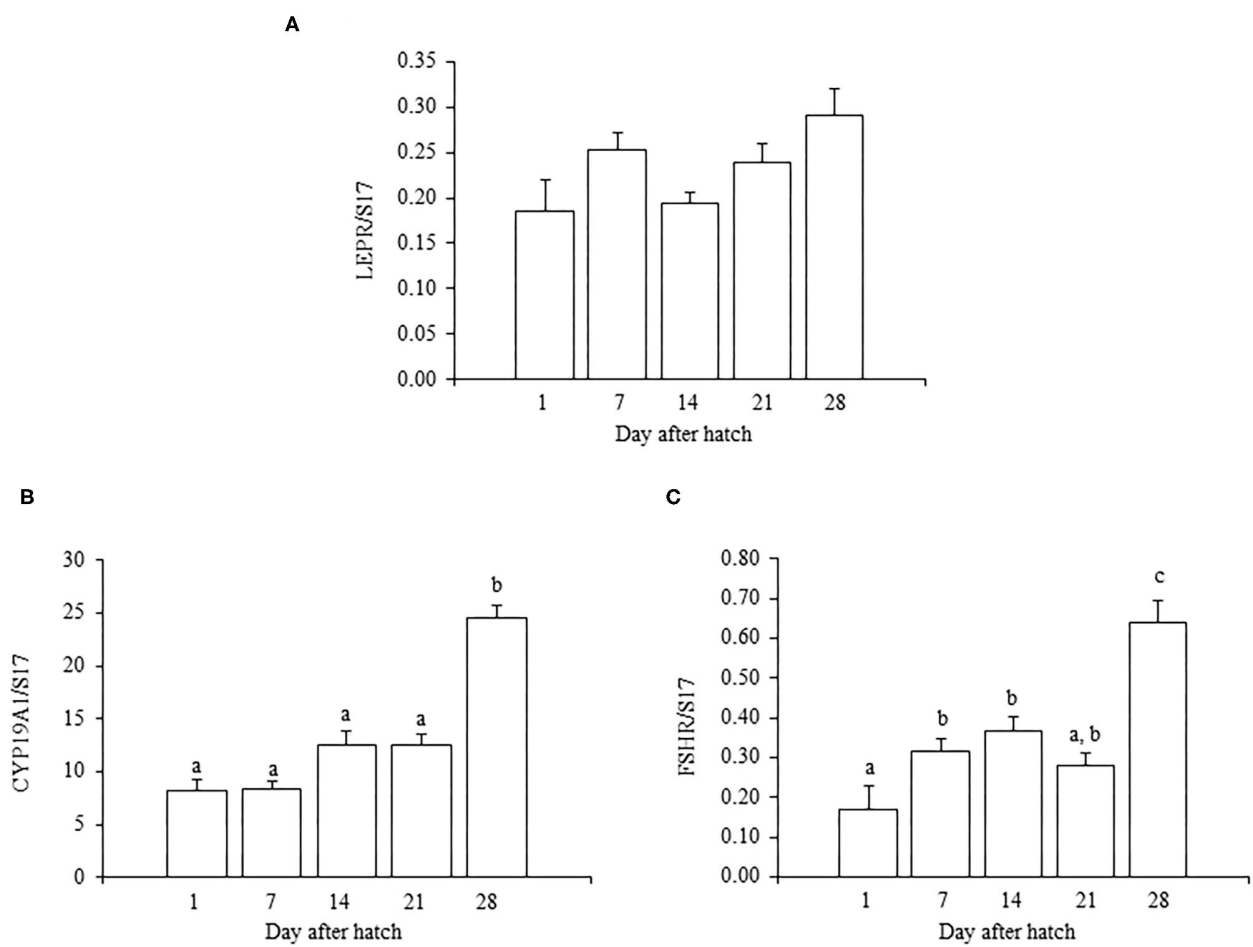
The mRNA expression of LEPR **(A)**, FSHR **(B)**, and CYP19A1 **(C)** in the ovary in growing juvenile chicks. The data represent the mean ± SEM (*n* = 4), and bars with different superscripts are significantly different.

In juvenile chicks, the mRNA expression of LEPR generally increased as they aged, but the expression at 28 days old was not significantly different from that at 1 day old (*P* = 0.055) (Figure 1A). In addition, the mRNA expression of LEPR in 7-day-old chicks was comparatively higher than that in 1- and 14-day-old chicks (Figure 1A). The mRNA expressions of FSHR and CYP19A1 gradually increased as chicks aged, and the expression of each reached a peak at 28 days old (Figures 1B,C). The FSHR expression in 28-day-old chicks was significantly higher than that on all other examined days (Figure 1B). The mRNA expression of CYP19A1 in 28-day-old chicks also differed significantly from the expression in all other ages (Figure 1C).

### mRNA Expression Profile of Genes in the HPG Axis of 7-Day-Old Chicks Injected With Leptin

**Figure 2:**
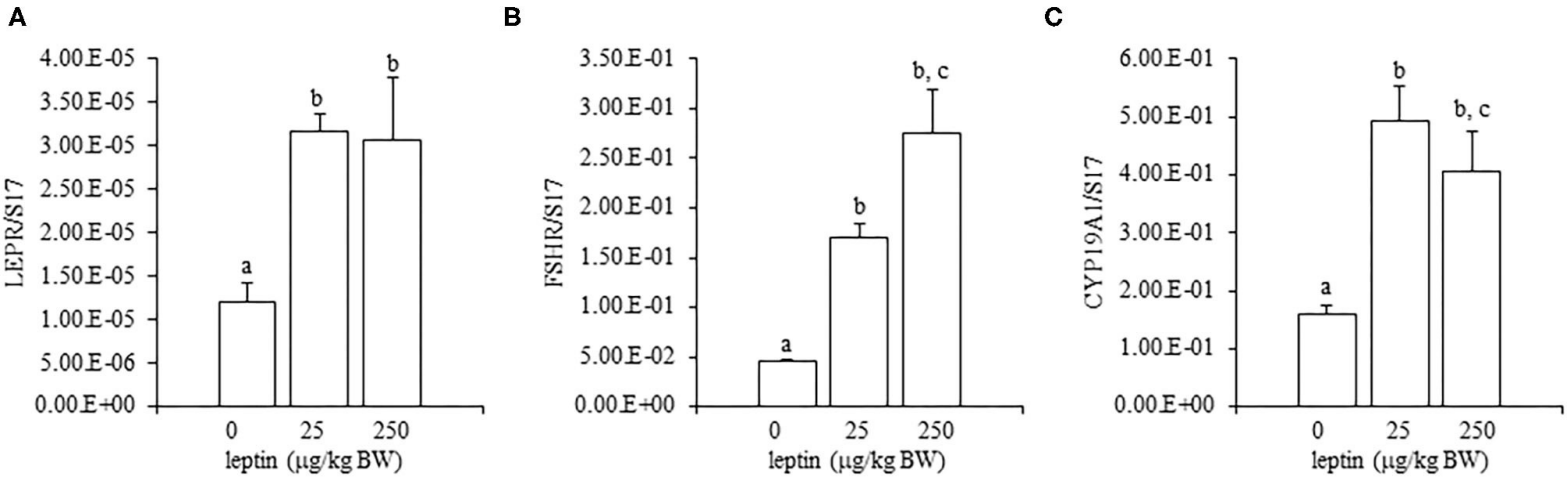
Ovarian mRNA expression of LEPR **(A)**, FSHR **(B)**, and CYP19A1 **(C)** at 24 h after leptin treatment in the 7-day-old chicks. Chicks were injected intraperitoneally (i.p.) with 0, 25, and 250 μg/kg body weight (BW) of recombinant mouse leptin (rmleptin). The data represent the mean ± SEM (*n* = 4), and bars with different superscripts are significantly different.

**Figure 3:**
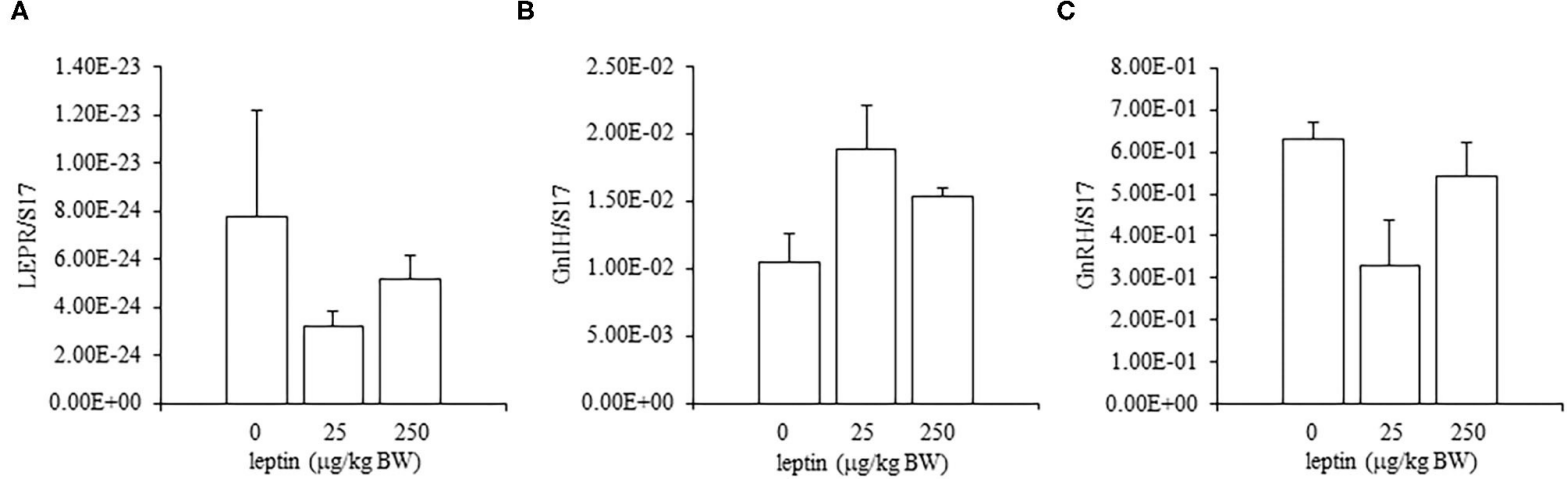
Hypothalamic mRNA expression of LEPR **(A)**, GnRH1 **(B)**, and GnIH **(C)** at 24 h after leptin administration in the 7-day-old chicks. Chicks were injected i.p. with 0, 25, and 250 μg/kg BW of rmleptin. The data represent the mean ± SEM (*n* = 6).

**Figure 4:**
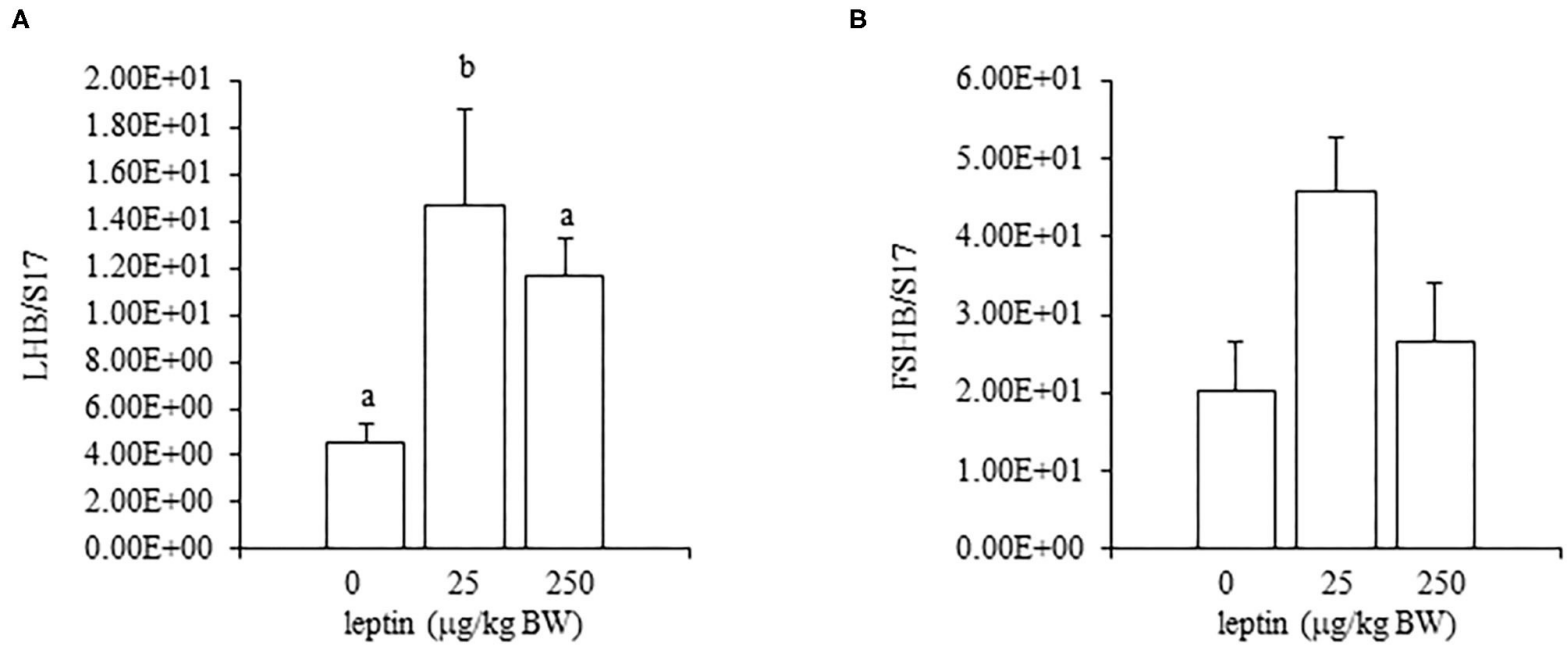
The mRNA expression of LHB **(A)** and FSHB **(B)** at 24 h after leptin treatment in the pituitary gland in the 7-day-old chicks. Chicks were injected i.p. with 0, 25, and 250 μg/kg BW of rmleptin. The data represent the mean ± SEM (*n* = 4–6), and bars with different superscripts are significantly different.

For ovarian LEPR, the administration of both 25 and 250 μg/kg BW leptin increased the mRNA expression significantly (*P* < 0.05; Figure 2A). The mRNA expression of FSHR increased significantly with leptin administration in a dose-dependent manner (*P* < 0.05; Figure 2B). The ovarian mRNA expression of CYP19A1 also increased significantly with the administration of both leptin doses (*P* < 0.05; Figure 2C). On the other hand, hypothalamic LEPR, GnRH1, and GnIH mRNAs were not changed significantly by leptin administration in 7-day-old chicks (Figure 3). In the pituitary gland, exogenous leptin did not affect the mRNA expression of FSHB, but the 25 μg/kg BW leptin treatment increased the mRNA expression of LHB significantly (Figure 4).

### mRNA Expression Profile of Genes in the HPG Axis of 28-Day-Old Chicks Injected With Leptin

**Figure 5:**
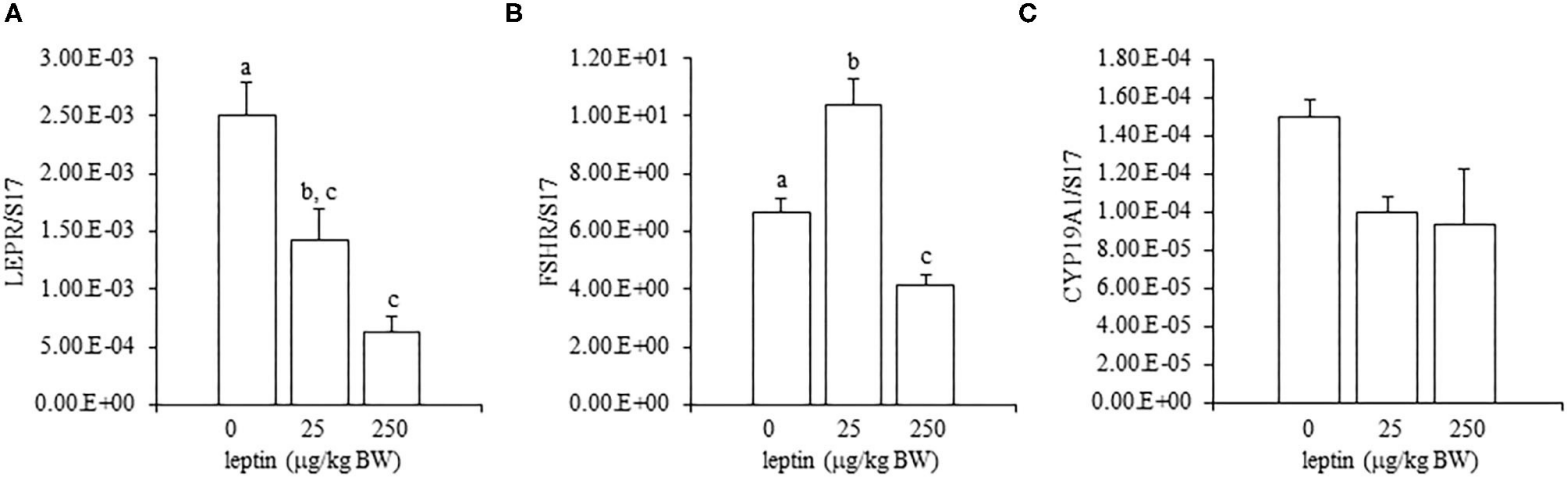
Ovarian mRNA expression of LEPR **(A)**, FSHR **(B)**, and CYP19A1 **(C)** at 24 h after leptin treatment in the 28-day-old chicks. Chicks were injected i.p. with 0, 25, and 250 μg/kg BW of rmleptin. Bars with different superscripts are significantly varied. The data represent the mean ± SEM (*n* = 6), and bars with different superscripts are significantly different.

**Figure 6:**
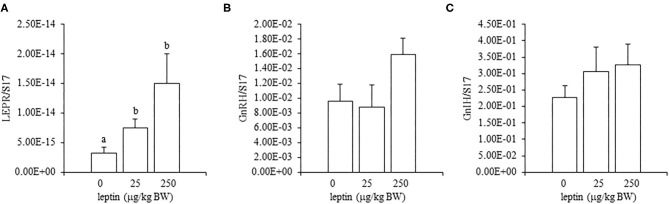
Hypothalamic mRNA expression of LEPR **(A)**, GnRH1 **(B)**, and GnIH **(C)** at 24 h after leptin administration in the 28-day-old chicks. Chicks were injected i.p. with 0, 25, and 250 μg/kg BW of rmleptin. The data represent the mean ± SEM (*n* = 6), and bars with different superscripts are significantly different.

**Figure 7:**
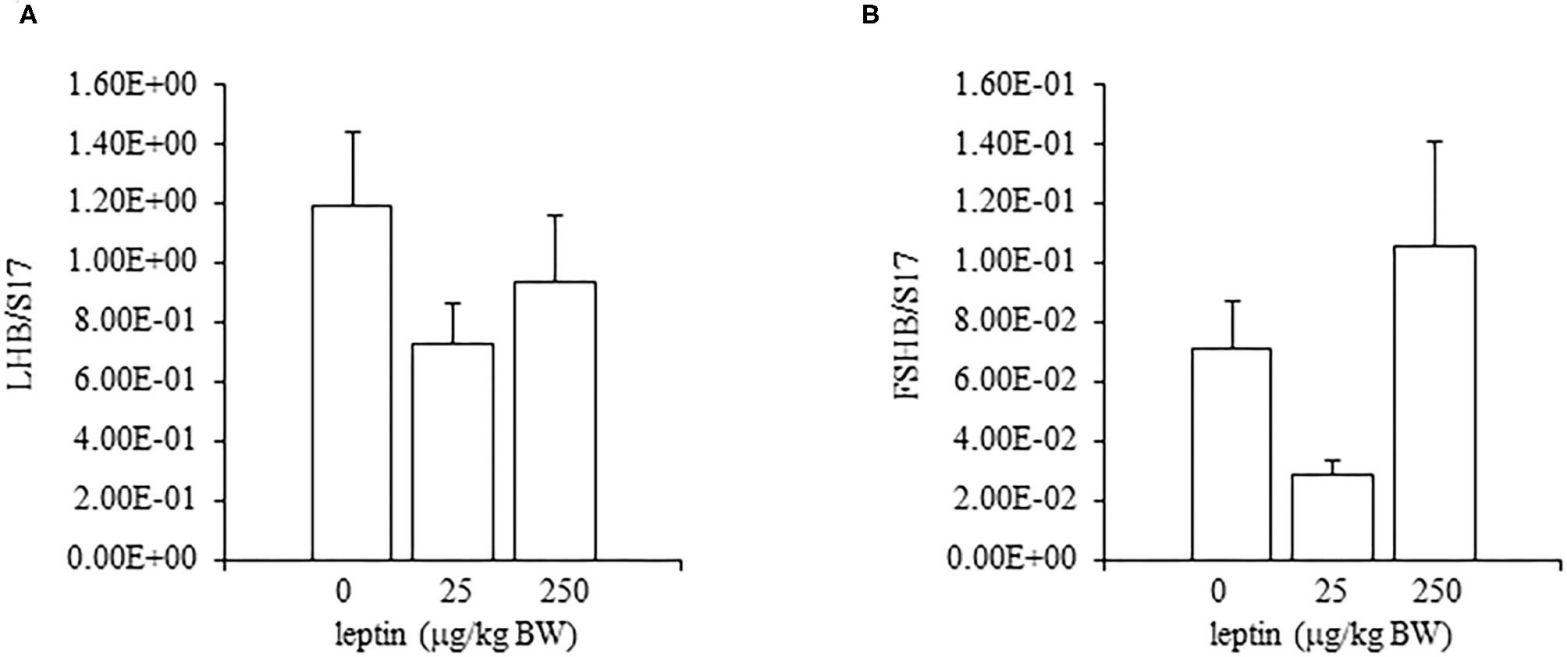
The mRNA expression of LHB **(A)** and FSHB **(B)** at 24 h after leptin treatment in the pituitary gland in the 28-day-old chicks. Chicks were injected i.p. with 0, 25, and 250 μg/kg BW of rmleptin. The data represent the mean ± SEM (*n* = 6). Each raw data are shown as a dot.

In the ovaries, the mRNA expression of LEPR decreased, in a dose-dependent manner, with leptin treatment (*P* < 0.05; Figure 5A). The mRNA expression of FSHR increased significantly with the lower dose of leptin (25 μg/kg BW; *P* < 0.05), whereas the higher dose of leptin (250 μg/kg BW) significantly reduced the mRNA expression of FSHR (Figure 5B). In contrast, leptin treatment had no effect on the mRNA expression of CYP19A1 in the ovary (Figure 5C). The hypothalamic mRNA expression of LEPR showed an increasing trend following leptin administration, but again, leptin had no effect on the hypothalamic mRNA expression of GnRH1 or GnIH in chicks of this age (Figure 6). In the pituitary gland, low doses of leptin tended to reduce both the mRNA expressions of FSHB and LHB in 28-day-old chicks, but these effects were not significant (Figure 7).

### Ovarian mRNA Expression of SOCS3 After Leptin Administration in 7- and 28-Day-Old Chicks

**Figure 8:**
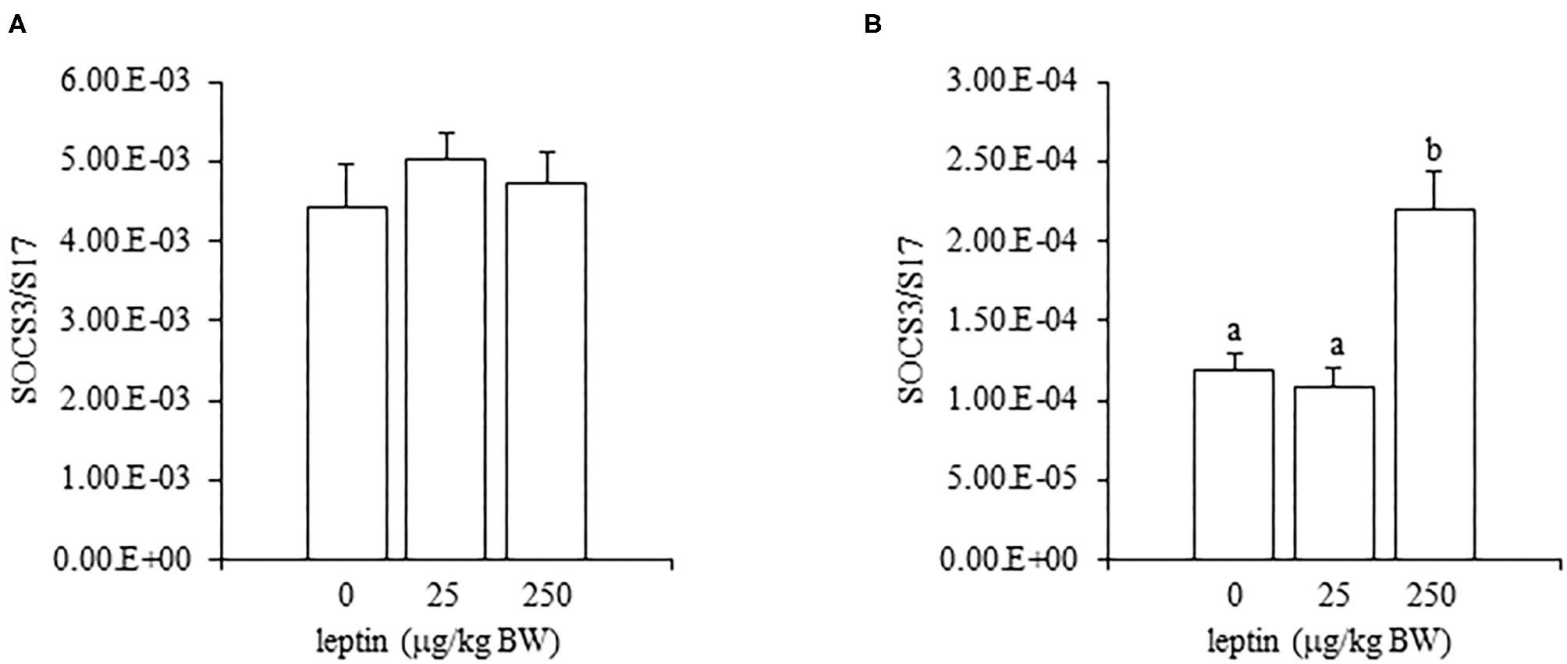
The mRNA expression of SOCS3 in the ovary at 24 h after leptin treatment in 7-day-old **(A)** and 28-day-old **(B)** chicks. Chicks were injected i.p. with 0, 25, and 250 μg/kg BW of rmleptin. The data represent the mean ± SEM (*n* = 4), and bars with different superscripts are significantly different.

Leptin administration had no effect on the ovarian mRNA expression of SOCS3 in 7-day-old chicks; however, a high dose of leptin (250 μg/kg BW) upregulated the mRNA expression of SOCS3 in 28-day-old chicks (Figure 8).

### Effects of Leptin on Serum Estradiol in 7- and 28-Day-Old Chicks

Table 2 shows serum estradiol concentrations 24 h after leptin administration in 7- and 28-day-old chicks. Serum estradiol levels significantly increased with leptin treatment in 7-day-old chicks (*P* < 0.05). However, in 28-day-old chicks, serum estradiol levels significantly decreased when the highest dose of leptin (250 μg/kg BW) was administered (*P* < 0.05).

**Table 2:** Serum estradiol concentration 24 h after leptin administration at 7- and 28-days old chicks.

	**Leptin (μg/kg BW)**		
	**0**	**25**	**250**
Day 7	11.18 ± 1.16^a^	15.38 ± 0.46^b^	15.78 ± 0.21^b^
Day 28	15.18 ± 0.44^a^	15.65 ± 0.42^a^	13.01 ± 0.57^b^

*BW, body weight. Different superscripts in same row are significantly different. Data represent the mean ± SEM (n = 5)*.

## Discussion

Hormones and biologically active substances in the HPG axis regulate gonadal function and development, either directly or indirectly. Leptin is also one of these factors that modulate ovarian development and folliculogenesis in vertebrates including birds. For instance, exogenous leptin administered to prepubescent females advances puberty in chickens (Paczoska-Eliasiewicz et al., 2006) and the *in ovo* administration of leptin causes early onset of puberty in Japanese quail (Lamošová et al., 2003).

In this study, we first analyzed the ontogenic mRNA expression of LEPR and ovarian functional markers, FSHR and CYP19A1, in the juvenile chicken ovary. The mRNA expression of LEPR generally increased with age in the ovary of juvenile chickens, and the expression at 28 days old was greater than at 1 or 14 days old (Figure 1A). However, there was a small, but not significant, anomalous increase in the mRNA expression of LEPR in 7-day-old chicks, which was greater than that observed at 14 days old (Figure 1A). It is reported that the juvenile ovary expresses significantly higher LEPR mRNA than that of the matured ovary in birds (Lei et al., 2014; Wang et al., 2016). During the same ontogenic process, the expression of the ovarian functional markers FSHR and CYP19A1 increased with the ages of chicks (Figures 1B,C) and both FSHR and CYP19A1 markedly increased between 21- and 28-day-old chicks in this study. This result is consistent with a recent finding that the FSHR expression increases in association with the progress of folliculogenesis in the post-hatch geese and ducks (Hu et al., 2021). Moreover, Y123F mouse in which 3-tyrosine residues in the long isoform of LEPR (LEPRb) were substituted into phenylalanine showed the decrease in serum estrogen level (Tu et al., 2018). In addition, the administration of FSH restored the mRNA expression of CYP19A1 in the Y123F mouse (Tu et al., 2018). Therefore, the change in FSHR and CYP19A may correlate an age-dependent contribution of leptin with follicle growth and development in the post-hatched chicken, and leptin may exert on this process. In the post-hatch development of the ovary, it is known that primary oocytes become organized into primordial follicles in the ovary at 7 days old (González-Morán, 2007, 2011). In addition, primordial follicles transit to primary follicles at 4 weeks (Johnson and Woods, 2007). Therefore, this study explored whether leptin had any role in modulating ovarian development at these critical points.

In the ovary of 7-day-old chickens, leptin dose-dependently increased the mRNA expressions of LEPR and FSHR, and leptin upregulated the mRNA expression of CYP19A1 (Figure 2). On the other hand, leptin did not have specific effects on GnIH and GnRH expression in the hypothalamus of 7-day-old chickens (Figure 3). Therefore, it is likely that hypothalamic factors do not have a specific role in regulating ovarian markers by leptin at this stage. Regarding the mRNA expression of gonadotropin in the pituitary gland, leptin significantly increased the mRNA expression of LHB but not the mRNA expression of FSHB (Figure 4). Plasma FSH remains low until puberty, but the LH level increases during the first week after hatching (Vanmontfort et al., 1995). The mRNA expression observed in this study may reflect the plasma level of gonadotropins at this age. Moreover, we found that leptin administration significantly increases the serum estradiol levels (Table 2). Estrogen is synthesized in the ovary of chick at the timing of primordial assembly (Yoshida et al., 1996) and promotes primordial follicle formation in chicks (Zhao et al., 2017). The primordial follicles are enclosed by a single layer of granulosa cells at this timing (González-Morán, 2011; Zhao et al., 2017). FSH is known to stimulate estrogen release from pre-hierarchical follicles (Hu and Zadworny, 2017), and the pre-hierarchical granulosa cells express LEPR (Hu et al., 2014; Wen et al., 2015). In addition, leptin alters sterol/steroidogenic genes in the goose granulosa cells (Hu et al., 2014). In pigeon, 3β-hydroxysteroid dehydrogenase (3βHSD) localized in the granulosa cell around follicles in late embryonic and early post-hatch chicks (Olea et al., 2020). Moreover, FSH promotes the assembly of primordial follicle (Guo et al., 2019a,b) and that increased 2–3 days after the increase in the mRNA expression of FSHR (Guo et al., 2019a). Therefore, in this study, the leptin-induced increase in FSHR may alter ovarian sensitivity to FSH and that may initiate early follicle development as well as steroidogenesis in chicken. In fact, our preliminary experiment found that leptin treatment for 2 days induced the assembly of primordial follicles to the ovarian cortex and increases follicle development (Supplementary Figure 1). We also found that leptin stimulation decreased the mRNA expression of caspase-3 in the ovary at the same age (Supplementary Figure 2), and this result is consistent with the observation that FSH inhibits apoptosis of ovarian cells in the ovary of 4-day-old chicks (Guo et al., 2019a).

Given the observed leptin-induced increase in LHB mRNA, it should be also noted that there is a temporary increase in plasma LH on day 7 after hatching (Sharp, 1975; Vanmontfort et al., 1995; Ellestad et al., 2011) and that LH treatment in chick embryos promotes the transition of oogonia to oocyte by 1 week of age, when the primordial follicle assembly takes place (González-Morán, 2007). Furthermore, in Japanese quail, the *in ovo* administration of leptin advances puberty (Lamošová et al., 2003). Taken together, these findings may suggest that leptin stimulates follicle development during early post-hatch days by LH and FSH. The role of leptin and LH in the ovarian development of chicks immediately after hatching requires further investigation.

In experiments involving 28-day-old chickens, leptin induced different effects on the HPG axis than those observed at 7 days. Neither the hypothalamic mRNA expressions (of LEPR, GnRH1, and GnIH) nor the pituitary gland mRNA expressions (of FSHB and LHB) were changed significantly by any leptin dose in 28-day-old chicks (Figures 6, 7). Since leptin did not modify hypothalamus–pituitary genes related to gonadal functions, it may not exert central control of the ovarian development at this age. In the ovaries of 28-day-old chickens, the administration of leptin downregulated the mRNA expression of LEPR, but it had no effect on CYP19A1 mRNA (Figures 5A,C). However, a lower dose of leptin (25 μg/kg BW) significantly increased the mRNA expression of FSHR, but a higher dose (250 μg/kg BW) reduced it (Figure 5B). Furthermore, serum estrogen levels were unaffected by lower leptin doses, but higher doses reduced them significantly (Table 2). Inhibitory response of ovary to high dose of leptin has been observed in fasted matured ducks. A high amount of leptin attenuated the recovery of FSHR expression and plasma estradiol (Song et al., 2009). By 28 days, primary follicles develops, and the theca layer begins to form by the lacunar channels (González-Morán, 2011). As described earlier, this dynamic may change the characteristics of ovarian cells. In this study, ovarian FSHR and CYP19A1 expression increased substantially from 21- to 28-days-old chicks. It may indicate that the responsiveness to leptin have changed according to the progress of ovarian development, because a high dose of leptin in the advanced stage (i.e., 28-days old) decreased the ovarian mRNA expression of LEPR (Figure 5) and increased the ovarian mRNA expression of SOCS3 (Figure 8), but such change was not observed in an earlier day (i.e., 7 days old). There is evidence that a higher dose of leptin downregulated the LEPR expression in the geese granulosa cells obtained from F1–F3 follicles *in vitro* (Hu et al., 2014) and that may support our hypothesis. However, it is necessary to clarify how hyperleptinemia causes a decrease in estrogen release and the reduction in FSHR mRNA in 28-day-old chicks.

This study revealed for the first time that leptin modulates the gene expression of ovarian development and folliculogenesis marker genes on specific ovarian-remodeling days in post-hatch chicks, and that may alter folliculogenesis and ovarian development toward puberty in chicken. This is in concordance with previous findings that exogenous leptin, which was administered at an early age, may accelerate sexual maturation in birds (Lamošová et al., 2003; Paczoska-Eliasiewicz et al., 2006). Furthermore, leptin may primarily act directly on the ovary in the juvenile days because this study revealed that leptin did not alter hypothalamic and pituitary gene expression, except LHB, at these ages, and the mRNA expression of leptin was found in the ovary in red jungle fowl (Seroussi et al., 2016). Recently, it was found that TGF-β1 increases apoptotic cells to restrain follicle formation in the ovary of 5-day-old chicks (Zhou et al., 2020). We also found in this study that the mRNA expression of an apoptotic marker, i.e., caspase-3, was modified by leptin (Supplementary Figure 2). In addition, autophagy may accelerate the transition from oocyte to primordial follicles in mice (Watanabe et al., 2020) and leptin induces autophagy in the chicken tissues (Piekarski et al., 2018). Therefore, further experiments on clarifying the relationship between leptin and programmed cell death may be valuable to better understand the primordial follicle formation and further ovarian development in chicken.

## Data Availability Statement

The original contributions presented in the study are included in the article/Supplementary Material, further inquiries can be directed to the corresponding author/s.

## Ethics Statement

The animal study was reviewed and approved by Ibaraki University Animal Care and Use Committee.

## Author Contributions

AHS, MO, and AS performed the experiments. MT and AA performed the technical support and the statistical analysis of the experiments. TO designed the experiment and wrote the manuscript. All authors contributed to the article and approved the submitted version.

## Conflict of Interest

The authors declare that the research was conducted in the absence of any commercial or financial relationships that could be construed as a potential conflict of interest.
