# Supplementary figures and images for: Leptin Modulates the mRNA Expression of Follicle Development Markers in Post-hatch Chicks in an Age-Dependent Manner
Source: Front Physiol. 2021 Jul 7;12:657527. doi: 10.3389/fphys.2021.657527 (PMC8293390; doi:10.3389/fphys.2021.657527)

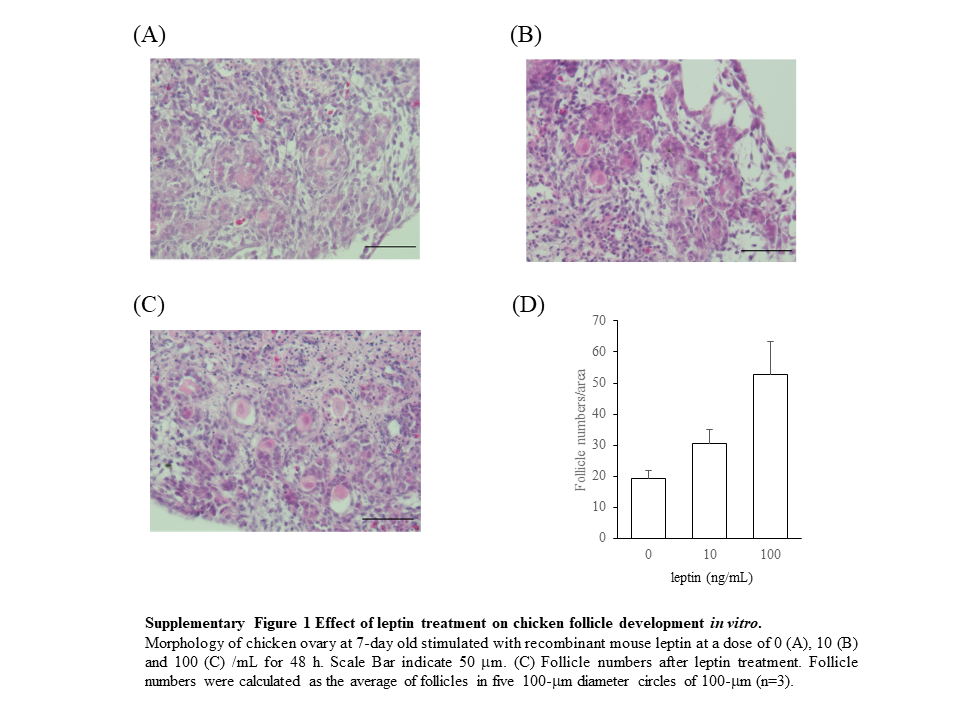

Supplement: Supplementary file 1 [file Image_1.TIF]

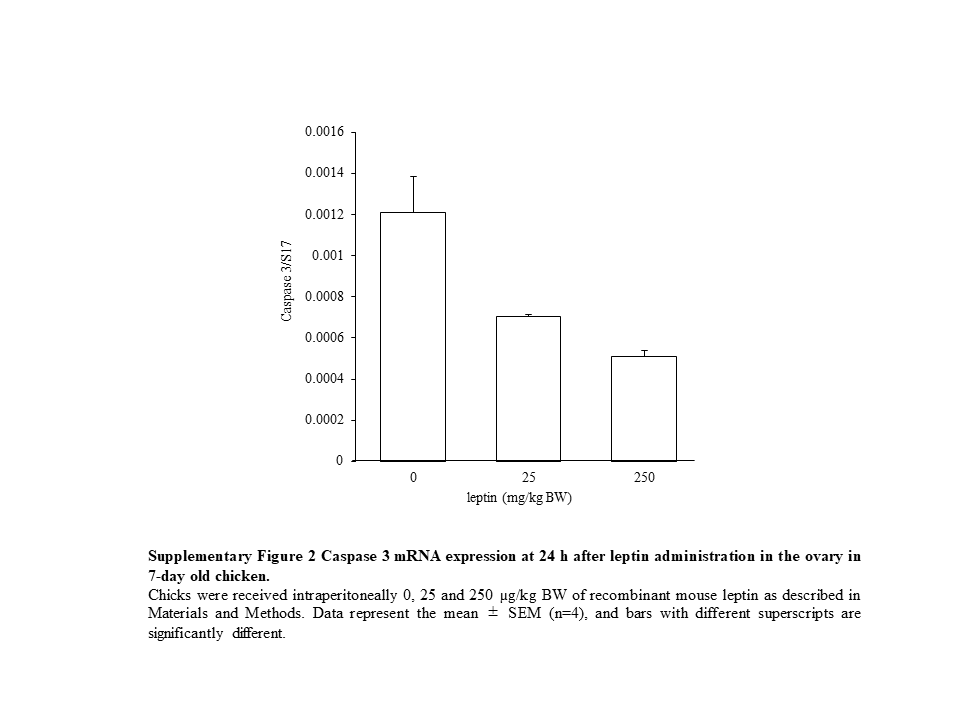

Supplement: Supplementary file 2 [file Image_2.TIF]
